# Antibiotic use for community-acquired pneumonia in neonates and children: WHO evidence review

**DOI:** 10.1080/20469047.2017.1409455

**Published:** 2018-05-23

**Authors:** Shrey Mathur, Aline Fuchs, Julia Bielicki, Johannes Van Den Anker, Mike Sharland

**Affiliations:** a Paediatric Infectious Disease Research Group, Institute for Infection and Immunity, St George’s University of London, London, UK; b Paediatric Pharmacology and Pharmacometrics, University Children’s Hospital Basel, Basel, Switzerland; c Division of Clinical Pharmacology, Children’s National Health System, Washington, DC, USA

**Keywords:** Pneumonia, bacterial, community-acquired pneumonia, antimicrobial resistance, AAD, antibiotic-associated diarrhoea, BNFc, British National Formulary for Children, BTS, British Thoracic Society, CAP, community-acquired pneumonia, CPS, Canadian Paediatric Society, EARS-Net, European Antimicrobial Resistance Surveillance Network, ESPID, European Society for Paediatric Infectious Diseases, GRADE, Grading of Recommendations Assessment, Development and Evaluation, IDSA, Infectious Diseases Society of America, IMCI, integrated management of childhood illness, PCV, pneumococcal conjugate vaccine, PIDS, Pediatric Infectious Diseases Society, RCPCH, Royal College of Paediatrics and Child Health, WHO, World Health Organization

## Abstract

**Background** Pneumonia is the most common cause of death in children worldwide, accounting for 15% of all deaths of children under 5 years of age. This review summarises the evidence for the empirical antibiotic treatment of community-acquired pneumonia in neonates and children and puts emphasis on publications since the release of the previous WHO Evidence Summary report published in 2014.

**Methods** A systematic search for systematic reviews and meta-analyses of antibiotic therapy for community-acquired pneumonia was conducted between 1 January 2013 and 10 November 2016.

**Results** The optimal dosing recommendation for amoxicillin remains unclear with limited pharmacological and clinical evidence. There is limited evidence from surveillance to indicate whether amoxicillin or broader spectrum antibiotics (e.g. third-generation cephalosporins) are being used most commonly for paediatric CAP in different WHO regions. Data are lacking on clinical efficacy in the context of pneumococcal, staphylococcal and mycoplasma disease and the relative contributions of varying first-line and step-down options to the selection of such resistance.

**Conclusion** Further pragmatic trials are required to optimise management of hospitalised children with severe and very severe pneumonia.

## Introduction

This review summarises the most up-to-date evidence for the empirical antibiotic treatment of community-acquired pneumonia (CAP) in neonates and children. For this update, special emphasis has been placed on publications since the release of the previous report *‘*Revised WHO Classification and Treatment of Pneumonia in Children at Health Facilities: Evidence Summaries’ in 2014 [1]. As the 2014 guideline was both recent and a major revision of guidance, this review summarises the recent literature and discusses emerging challenges.

CAP refers to pneumonia acquired in the community. Pneumonia accounts for 15% of all deaths of children <5 years of age and is the single largest infectious cause of death in children worldwide. In HIV-uninfected children, pneumococcal infection is responsible for around 11% of all deaths in this age group [1]. Nearly, 1 in 500 children under the age of 5 years is hospitalised each year with CAP [2]. However, only 54% of children with symptoms of pneumonia are taken outside the home for care [3]. Pneumonia affects children and families everywhere, but is most prevalent in South Asia and sub-Saharan Africa [1]. There are signs of progress in the 75 countries included in ‘Countdown to 2015’ [3]. In this group, the number of deaths from pneumonia in children under 5 has declined from 21% in 2000 to 16% in 2015 [3]. Nonetheless, CAP remains an issue of profound economic and social importance to children and communities worldwide.

Aetiological studies of CAP in children are complicated by the low yield of blood cultures, inadequate sputum specimens and infrequent work-up with lung aspiration and broncho-alveolar lavage. Quantification of aetiology is further complicated by limited microbiological work-up in the community, seasonality, mixed infections and viruses and commensal bacteria in samples.

*Streptococcus pneumoniae* is widely considered to be the leading cause of CAP, though proportions vary by region. It is responsible for about one-third of radiologically confirmed pneumonia in children aged <2 years. *S. pneumoniae* is commonly found in asymptomatic nasopharyngeal carriage. The asymptomatic carriage state is responsible for much of the transmission within populations, such as day-care centres [4]. *Haemophilus influenzae* type b (Hib) is a major pathogen, though proportions vary regionally and with vaccine coverage [5]. Pneumonia caused by *Mycoplasma pneumoniae* is considered an atypical bacterial pneumonia because of its different course, radiological findings and treatment. Active population-based surveillance for CAP was undertaken in hospitals in three American cities [6]. The annual incidence of hospitalisation for pneumonia was 15.7 cases per 10,000 children (95% CI 14.9–16.5). The annual incidence of *M. pneumoniae* was 1.4/10,000 (95% CI 1.2–1.6) and of *S. pneumoniae* was 0.5/10,000 (95% CI 0.4–0.6) [6]. Less commonly, severe infection is caused by *Staphylococcus aureus*, especially following influenza. Fungal infection by *Pneumocystis jiroveci* (PJP) is particularly important in young children with AIDS. Furthermore, children with milder atypical pathogens may recover without antibiotic intervention. Causative pathogens also vary with age. Overall, viruses alone are a cause in younger children, in up to 50%. When a bacterial cause is found in older children, it is most commonly *S. pneumoniae*, followed by *M. pneumoniae* [7].

One-third of cases of CAP are a mixed infection with viruses and bacteria [7]. Viruses commonly associated with CAP are respiratory syncytial virus (RSV), para-influenza and influenza. Other viruses isolated in children with pneumonia include adenovirus, rhinovirus, herpes simplex virus, enteroviruses, human metapneumovirus, human bocavirus and coronavirus. Overall, viruses account for 30–67% of childhood CAP and are more frequently identified in children aged <1 year than in those aged >2 years [7].

## Current WHO guidelines and rationale

The ‘Revised WHO Classification and Treatment of Pneumonia in Children at Health Facilities: Evidence Summaries’ was published in 2014 [1]. The revision integrated input from two consultations which used the GRADE approach (Grading of Recommendations Assessment, Development and Evaluation): the 2010 WHO Recommendations on the Management of Diarrhoea and Pneumonia in HIV-infected Infants and Children: Integrated Management of Childhood Illness (IMCI) and the 2012 Recommendations for Management of Common Childhood Conditions, Evidence for Technical Update of Pocket Book Recommendations. The revisions include updating the classification of pneumonia severity and changing the recommendation for first-line antibiotics [1].

The 2014 guidance reclassified CAP requiring treatment at a healthcare facility into three categories: very severe pneumonia, severe pneumonia and non-severe pneumonia. The new approach was designed to simplify the management of pneumonia at the outpatient level, reduce the number of referrals for hospitalisation and achieve better treatment outcomes.

Very severe pneumonia is defined as cough or difficulty breathing plus any of the following: central cyanosis; inability to breastfeed, drink, or vomiting everything; convulsions, lethargy, or unconsciousness; and severe respiratory distress. Severe pneumonia is defined as cough or difficulty breathing and one of the following: lower chest-wall indrawing; nasal flaring; grunting (in young infants) with no signs of very severe pneumonia, especially if <2 months of age.

Non-severe pneumonia is defined as cough or difficulty breathing accompanied by tachypnoea (respiratory rate ≥50 breaths/minute in infants aged 2–11 months, ≥40 breaths/minute in children aged 12–59 months) with no signs of severe or very severe pneumonia, especially if aged ≥2 months. The WHO definition of treatment failure includes development of signs of severe or very severe pneumonia and persistently raised respiratory rate at 72 h (48 h in areas with a high prevalence of HIV).

Previous to the 2014 revision, four treatment categories were defined for CAP. Children with ‘fast breathing’ pneumonia were treated with oral cotrimoxazole. Children with ‘chest indrawing’ pneumonia were referred to a healthcare facility and treated with injectable penicillin/ampicillin. As a result of new evidence, the 2014 revision preferred oral amoxicillin to oral cotrimoxazole for the treatment of fast-breathing pneumonia and was equivalent to injectable penicillin/ampicillin in cases of chest-indrawing pneumonia. Since both fast-breathing and chest-indrawing pneumonias were now best treated with amoxicillin, classifications were also revised. The new classification was revised to include only two categories of pneumonia: ‘pneumonia’ with fast breathing and/or chest indrawing, which requires home therapy with oral amoxicillin, and ‘severe pneumonia’, pneumonia with any general danger sign, which requires referral and injectable antibiotic therapy.

## Summary of international guidelines

Recently published international clinical practice guidelines were also reviewed. These included clinical practice guidelines in the British National Formulary for Children (BNFc) [8], the Royal College of Paediatrics and Child Health (RCPCH) [9], the European Society for Paediatric Infectious Diseases (ESPID) [10], the Canadian Paediatric Society (CPS) [11], the British Thoracic Society (BTS) [7] and the Pediatric Infectious Diseases Society (PIDS) [12]. These have been summarised in Table 1.

**Table 1.** Comparison of international guidance for antibiotic therapy of community-acquired pneumonia in children.

Guideline	Last update	Recommendation
British National Formulary for Children (BNFc) [8]	2016	• Benzylpenicillin with gentamicin for neonatal sepsis of all causes • For children aged 1 month to 18 years, oral amoxicillin is recommended as first-line for CAP and clarithromycin is recommended if there is no response to treatment • For suspected staphylococcal infection, oral amoxicillin and flucloxacillin or amoxicillin clavulanate alone are recommended. • In complicated pneumonia or if oral administration is not possible, treatment for 7 days with intravenous amoxicillin or amoxicillin clavulanate or cefuroxime or cefotaxime is recommended. • For children aged 1 month–18 years who are allergic to penicillin, clarithromycin for 7 days is recommended
RCPCH/ESPID Manual of Childhood Infections, ‘Blue Book’ [9]	2016	• For children aged <5 years, oral amoxicillin for a standard course of 5 days is the first-choice antibiotic. • Macrolides are recommended if either *M. pneumoniae* or *Chlamydia pneumoniae* is suspected. • Intravenous antibiotics for severe pneumonia include penicillin/amoxicillin, amoxicillin clavulanate, cefuroxime and cefotaxime/ceftriaxone
British Thoracic Society [7]	2011	• Amoxicillin is first choice for oral antibiotic therapy in all children because it is effective against the majority of pathogens, is well tolerated and is cheap Alternatives are amoxicillin clavulanate, cefaclor, erythromycin, azithromycin and clarithromycin • Macrolide antibiotics may be added at any age if there is no response to first-line empirical therapy • Macrolide antibiotics should be used if either mycoplasma or *C. pneumoniae* is suspected or in very severe disease. • Amoxicillin clavulanate is recommended for pneumonia associated with influenza
Canadian Paediatric Society [11]	2015	• Oral amoxicillin is recommended for outpatients with lobar or broncho-pneumonia. • Patients who require hospitalisation but do not have a life-threatening illness should be commenced empirically on intravenous ampicillin. • Empirical therapy with a third-generation cephalosporin is recommended for children who experience respiratory failure or septic shock associated with pneumonia. • Ceftriaxone or cefotaxime are recommended for β-lactamase-producing *H. influenzae* and high-level penicillin-resistant pneumococcus. • For rapidly progressing multilobar disease or pneumatocoeles, the addition of vancomycin is suggested empirically with de-escalation to ampicillin with subsequent oral amoxicillin. • Vancomycin is recommended for empyema owing to *S. aureus*. • If *S. pneumoniae* is detected in blood or respiratory secretions and is penicillin-susceptible, treatment with either intravenous ampicillin or penicillin is recommended, followed by oral therapy with amoxicillin. • Treatment of *M. pneumoniae* and *C. pneumoniae* is azithromycin for 5 days. Doxycycline for children aged ≥8 years
ESPID [10]	2012	• For children <1 month, ampicillin/amoxicillin and gentamicin are recommended empirically. If *Listeria monocytogenes* or *enterococcus* is suspected, ampicillin with an alternative of a cephalosporin is recommended. Third-generation cephalosporins should be avoided in neonates because of the risk of candidiasis. For critically ill patients, an anti-staphylococcal penicillin and clindamycin or vancomycin are recommended. • For children aged 1–3 months, β-lactam antibiotics with anti-staphylococcal penicillin for the critically ill are recommended. In children with no fever or severe cough, *C. trachomatis* and *Bordetella pertussis* should be suspected and treated with macrolides. • For children aged 3 months to 5 years, penicillin G or aminopenicillins, e.g. amoxicillins are recommended to ensure adequate cover of *S. pneumoniae* and more atypical pathogens in this age group. For unimmunised children, treatment with amoxicillin clavulanate or a third-generation cephalosporin is recommended. Second-generation cephalosporins may be used in areas of low penicillin resistance. • For atypical pathogens, combined therapy with β-lactams (e.g. amoxicillin clavulanate) and macrolides (e.g. clarithromycin) are recommended as well as anti-staphylococcal antibiotics for the critically ill
IDSA/PIDS [12]	2011	• For children under 5 years of age, the guidelines recommend amoxicillin, amoxicillin clavulanate for presumed bacterial pneumonia and macrolides (azithromycin, clarithromycin or erythromycin) for presumed atypical pathogens. • For children over 5 years of age, amoxicillin, amoxicillin clavulanate and a macrolide can be added. The IDSA/PIDS guidelines recommend doxycycline for children >7 years

## Methods

A systematic search for systematic reviews and meta-analyses of antibiotic therapy for CAP published in English between 1 January 2013 to 10 November 2016 was undertaken. MEDLINE, the Cochrane Database for Systematic Reviews and ClinicalTrials.gov were searched. The search strategy of databases focused on clinical trials, controlled clinical trials, reviews or systematic reviews in all children (0–18 years). The search was conducted on 10 November 2016 combining MeSH and free-text terms ‘community-acquired infections’, ‘pneumonia, bacterial’, ‘community-acquired pneumonia’, ‘antibiotics’ and ‘anti-bacterial agents’. PubMed was searched for relevant guidelines. Titles and abstracts, full texts and subsequent data abstraction were screened independently, followed by consensus discussion.

## Results

### On-going clinical trials

Several clinical trials of antibiotics for CAP are registered on ClinTrialsGov.

A study in Beijing Children’s Hospital (NCT02775968) is investigating the population pharmacokinetics of cephalosporins and macrolide antibiotics for CAP in children, aiming to correlate it with treatment effectiveness and the incidence of adverse effects [13]. The study commenced in August 2016 with an estimated enrollment of 750 children and a completion date of October 2022.

A phase 2/3, randomised, open-label, active control, multi-centre study (NCT02605122) to assess the safety and efficacy of solithromycin in children and adolescents with CAP is being conducted under the sponsorship of Cempra Inc. [14]. Solithromycin will be compared with the standard of care for an estimated enrollment of 400 patients. The study commenced in March 2016 with an estimated completion date of January 2018.

A Canadian randomised, controlled, double-blind, non-inferiority clinical trial (NCT02380352) will determine whether 5 days of high-dose amoxicillin leads to comparable rates of early clinical cure compared with 10 days of high-dose amoxicillin for previously healthy children with mild CAP [15]. In the experimental arm, patients will be given 5 days of amoxicillin 90 mg/kg/day in three divided doses, followed by 5 days placebo three times a day. The active comparator arm will be given 5 days amoxicillin 90 mg/kg/day in three divided doses, followed by alternate formulation 5 days amoxicillin 90 mg/kg/day in three divided doses. The estimated enrollment for the study is 270 patients and it commenced in March 2016 with a completion date of May 2018.

The National Institute of Allergy and Infectious Diseases (NIAID) is sponsoring a multi-centre, randomised, double-blind, placebo-controlled, superiority clinical trial (NCT02891915) to test the effectiveness of short (5-day) *vs* standard (10-day) course therapy in children diagnosed with CAP and initially treated in outpatient clinics, urgent care facilities and emergency departments [16]. The primary objective is to compare the composite overall outcome (Desirability of Outcome Ranking, DOOR) in children with CAP aged 6–71 months assigned to a strategy of short course (5 days) *vs* standard course (10 days) outpatient β-lactam therapy at Outcome Assessment Visit 1 (Study Day 8 ± 2 days). The study commenced in October 2016 and the completion date is March 2019 with an estimated enrollment of 400 patients.

A Malaysian trial (NCT02258763) in children hospitalised with pneumonia is being conducted to determine whether an extended duration of oral antibiotics (10 days) is better for improving clinical outcomes than a shorter duration (3 days) of antibiotics [17]. Patients in the experimental arm will receive amoxicillin-clavulanate 22.5 mg/kg/dose bd for 10 days, while the comparator arm will receive amoxicillin-clavulanate 22.5 mg/kg/bd for 3 days followed by another 7 days of placebo given at the same dose and frequency. The study began in November 2014, aiming to enrol 300 patients, and the estimated completion date is December 2018.

Two clinical trials investigating amoxicillin in childhood pneumonia are being conducted in Malawi. In a trial (NCT02760420) sponsored by Save the Children, the effectiveness of no antibiotic treatment for fast-breathing CAP is being compared with amoxicillin therapy [18]. Patients in the placebo arm will be given 250 mg of placebo (dispersible tablet) in two divided doses based on age bands (500 mg/day for children 2–12 months, 1000 mg/day for children 12 months to 3 years, and 1500 mg/day for children 3–5 years of age). The active comparator arm will receive 3 days of 250 mg amoxicillin, dispersible tablet (DT) in two divided doses based on age bands (500 mg/day for children 2–12 months, 1000 mg/day for children 12 months to 3 years, and 1500 mg/day for children 3–5 years). The estimated enrollment is 2000 patients with the study running from June 2016 to September 2018.

In the same setting, another trial (NCT02678195) will compare 3 *vs* 5 days of treatment for chest-indrawing pneumonia [19]. The experimental arm will receive 3 days of amoxicillin and 2 days of placebo while the comparator arm will receive 5 days of amoxicillin. The study aimed to run from March 2016 to August 2018 with an estimated enrollment of 2000 patients.

A one-arm safety intervention (NCT02878031) in Nigeria will evaluate the role of community management of chest-indrawing pneumonia with oral amoxicillin [20]. The primary objective is to assess whether community health workers can safely and appropriately manage chest-indrawing pneumonia in children aged 2–59 months and refer children with danger signs. The aim was to include approximately 308 children aged 2–59 months with chest-indrawing pneumonia and the study was conducted between October 2016 and July 2017.

In a double blind efficacy study entitled RETAPP (NCT02372461), investigators based at Aga Khan University, Karachi compared standard amoxicillin treatment with placebo in poor urban slum settings in South Asia [21]. The study ran from November 2014 to July 2017 with an enrolment of 2500 patients.

Investigators in the United Kingdom are initiating a multi-centre, randomised, double-blind placebo-controlled 2 × 2 factorial non-inferiority trial of amoxicillin dosage and duration in paediatric CAP (CAP-IT) (ISRCTN76888927) [22]. The efficacy, safety and impact on antimicrobial resistance related to the duration and dosage of amoxicillin will be assessed in children aged 1–5 years presenting to the Emergency Room or Paediatric Assessment Unit with a clinical diagnosis of CAP in whom the decision has been made to treat with antibiotics. Participants will be randomised to four treatment groups: shorter course and lower dose (3 days of 35–50 mg/kg/day), longer course and lower dose (7 days of 35–50 mg/kg/day), shorter course and higher dose (3 days of 70–90 mg/kg/day), and longer course and higher dose (7 days of 70–90 mg/kg/day). They expect to recruit 2400 over the 2 years from March 2016 to May 2018.

### New antibiotics

Ceftaroline fosamil is a broadspectrum cephalosporin antibiotic with activity against many bacteria, including *S. pneumoniae* (both penicillin-non-susceptible and multi-drug-resistant strains) and *S. aureus* (including methicillin-resistant *S. aureus*) [23]. In a phase 2/3 study (NCT01530763), 160 paediatric patients hospitalised with CAP received either intravenous ceftaroline fosamil or ceftriaxone in a randomised, active-controlled, observer-blinded clinical trial. The effectiveness of ceftaroline fosamil was similar to that of ceftriaxone, with high clinical cure rates at test of cure in the modified intention-to-treat population (94/107; 88% and 32/36; 89%, respectively). Three documented *S. aureus* infections were successfully treated in the ceftaroline group, including one caused by methicillin-resistant *S. aureus*. In the phase 4 study (NCT01669980), the safety and effectiveness of ceftaroline fosamil in children was evaluated in a multi-centre, randomised, observer-blinded, active-controlled trial[24]. Ceftaroline fosamil was compared with intravenous ceftriaxone plus vancomycin in patients aged between 2 months and 17 years with complicated CAP. Clinical response rates in the modified intention-to-treat population were 52% (15/29 patients) in the ceftaroline fosamil group and 67% in the comparator group (6/9); clinical stability at Study Day 4 was 21% (6/29) and 22% (2/9), respectively. Ceftaroline fosamil was well tolerated and the clinical response rates were similar to that of ceftriaxone plus vancomycin.

### New interventions

Several interventions are complementary to antibiotic therapy for the management of CAP. Successful efforts are being made to integrate management and global vaccination campaigns.

The WHO Integrated Global Action Plan for the Prevention and Control of Pneumonia and Diarrhoea (GAPPD) aims to address protection, prevention and treatment of pneumonia and diarrhoea through integrated programmes in low- and middle-income countries (LMIC) [25]. Several identified interventions are specific to pneumonia, e.g. reduced household air pollution; pneumococcal conjugate vaccine (PCV), Hib and pertussis vaccination; and oxygen therapy when available. However, several interventions have been identified which are complementary for both pneumonia and diarrhoea. These are categorised as protect (breastfeeding promotion and support, adequate complementary feeding), prevent (measles vaccination, handwashing with soap, prevention of HIV) and treat (improving care-seeking behaviour and referral, improved case management at community and health facility levels, and continued feeding).

In the 2011–2015 strategic period, the Global Alliance for Vaccines and Immunization (GAVI) introduced PCV to 51 countries including Bangladesh, Cambodia, Eritrea, Guinea Bissau, Lesotho, Nepal, Solomon Islands and Uzbekistan [26]. To date, it is estimated that, with GAVI support, over 76 million children have received PCV. However, coverage with the third dose of vaccine was only 35% in 2015.

### New efficacy data

The KEMRI–Wellcome Trust Collaborative Research Programme conducted a trial (NCT01399723) to assess whether clinical outcome following initial treatment with oral amoxicillin for severe pneumonia is as effective as the current standard benzyl penicillin [27]. The open-label, multi-centre, randomised controlled non-inferiority trial of the treatment of severe pneumonia recruited 527 children aged 2–59 months in six Kenyan hospitals. The children were randomised to receive amoxicillin or benzyl penicillin and were followed up for the primary outcome of treatment failure at 48 h. The treatment failed in 20 of 260 (7.7%) and 21 of 261 (8.0%) patients in the amoxicillin and benzyl penicillin arms, respectively (RD −0.3%, 95% CI −5.0 to 4.3%), confirming the non-inferiority of amoxicillin to benzylpenicillin.

In the IndiaCLEN multi-centre trial (NCT01386840), the safety and efficacy of oral amoxicillin for severe pneumonia at home or in hospital were compared [28]. This open-label, multi-centre, prospective, two-arm, randomised clinical trial in children aged 3–59 months with severe pneumonia aimed to determine the differences in treatment failure between a 7-day course of oral amoxicillin for the first 48 h in hospital and being sent home on the same treatment after enrolment. A total of 1118 children were enrolled and randomised to the home (*n* = 554) or hospital group (*n* = 564). Overall treatment failure rate was 11.5% (per protocol analysis) [28]. In the intention-to-treat analysis, treatment was significantly more likely to fail in the hospital group than in the home group. Death rates at 7 or 14 days did not differ significantly (RD 0.0%, 95% CI −0.5 to 0.5). The median total treatment cost was INR 399 for the home group and INR 602 for the hospital group (*p* < 0.001) for the same 5% failure rate after 7 days of treatment in the random sub-sample. Home-based treatment with oral amoxicillin was equivalent to hospital treatment for the first 48 h in children with chest-indrawing pneumonia and was less expensive.

A Cochrane review examined antibiotics for CAP in children and made recommendations for countries with high case fatalitie rates owing to pneumonia in children without underlying morbidities and where point-of-care tests for identifying aetiological agents for pneumonia were not available [29]. Twenty-nine trials comparing multiple antibiotics to which 14,188 children had been enrolled were included. For non-severe CAP in ambulatory settings, amoxicillin compared with co-trimoxazole had similar failure rates (OR 1.18, 95% CI 0.91–1.51) and cure rates (OR 1.03, 95% CI 0.56–1.89). In children with severe pneumonia without hypoxaemia, oral antibiotics (amoxicillin/co-trimoxazole) compared with injectable penicillin had similar failure rates (OR 0.84, 95% CI 0.56–1.24), hospitalisation rates (OR 1.13, 95% CI 0.38–3.34) and relapse rates (OR 1.28, 95% CI 0.34–4.82). In very severe CAP, death rates were higher in children receiving chloramphenicol than in those receiving penicillin/ampicillin plus gentamicin (OR 1.25, 95% CI 0.76–2.07). Based on these findings, amoxicillin was recommended over co-trimoxazole for patients with CAP in ambulatory settings, with amoxicillin clavulanate and cefpodoxime as alternative second-line drugs. Oral amoxicillin was recommended for children in an ambulatory setting with severe pneumonia without hypoxaemia. For children hospitalised with severe and very severe CAP, penicillin/ampicillin plus gentamicin was superior to chloramphenicol. The other alternative drugs for such patients are amoxicillin clavulanate and cefuroxime.

A meta-analysis of trials in LMICs was undertaken to determine the most suitable antibiotic therapy for treating pneumonia (very severe, severe and non-severe) and examined type of drug, duration of illness and combination therapy [30]. Randomised controlled trials and quasi-RCTs that assessed the route, dose, combination and duration of antibiotics in the management of WHO-defined very severe/severe/non-severe CAP were included. Study participants included children aged 2–59 months with CAP. Twenty-two studies which enrolled 20,593 children were included in meta-analyses. A combination of penicillin/ampicillin and gentamicin was effective for very severe pneumonia, and oral amoxicillin was as effective as other parenteral antibiotics. Oral amoxicillin was also found to be effective in non-severe pneumonia. The review further found that a short 3-day course of antibiotics was as beneficial as 5-day course for non-severe pneumonia in children aged 2–59 months.

A systematic review and expert survey review identified several candidate predictors of oral antibiotic failure not currently used in childhood pneumonia referral algorithms; they included excess age-specific respiratory rate, young age, abnormal oxygen saturation and moderate malnutrition in children aged 2–59 months in resource-limited settings with WHO-defined non-severe pneumonia (fast breathing for age and/or lower chest-wall indrawing without danger signs) [31] . In nine studies which met the inclusion criteria, oral antibiotic failure rates ranged between 7.8 and 22.9%. Six studies found excess age-adjusted respiratory rate — defined either as WHO-defined very fast breathing for age or 10–15 breaths/min faster than normal WHO age-adjusted thresholds (<50 breaths/min for ages 2–11 months and <40 breaths/min for ages 12–59 months — and four studies reported young age to be predictive of failure of oral antibiotics [31].

The question of the *M. pneumoniae* spectrum, specifically macrolide use in CAP, is commonly encountered in paediatric practice. A meta-analysis of children with community-acquired lower respiratory tract infection treated specifically for *M. pneumoniae* was undertaken [32]. Sixteen articles detailing 17 studies were included. Several low-quality studies found a reduction in fever duration but the clinical impact of this effect was unclear. Meta-analysis of five randomised controlled trials showed a pooled risk difference of 0.12 (95% CI 0.04–0.20) favouring treatment with macrolides, tetracyclines or quinolones class antibiotics which was not statistically significant. Overall, the authors considered that there is insufficient evidence to support any conclusion about the efficacy of macrolides for *M. pneumoniae* CAP in children. Future studies should highlight the potential for confounding mixed infections, timing of intervention relative to symptom onset, and testing modalities that include a combination of serology and polymerase chain reaction assays.

## Discussion

The high prevalence of CAP in children means that clinicians and public health experts face on-going challenges in prescribing antibiotics for children. These challenges frame the appraisal of evidence and guide antibiotic choice.

Antibiotic dosing plays an important role in adverse events and, in turn, compliance with treatment. Antibiotic-associated diarrhoea (AAD) is a well-recognised adverse reaction to amoxicillin. A review of reported rates of AAD following oral penicillin treatment in paediatric clinical trials quantified the evidence and elucidated the dearth of strong evidence in this field [33]. Of 7729 paediatric patients, 17.9% had AAD. For amoxicillin, the pooled rate in six studies was 8.1% (range 1.87–17.5%). However, there was no demonstrable correlation between the dose of amoxicillin and the rate of AAD. Importantly, there was an association between age and diarrhoea related to oral penicillin. Younger children aged 1 month to 2 years experienced higher rates of ADD (18%) than children aged 2–7 (4%) and those older than 7 years (2%). While it is important to consider AAD, the precise mechanism and robust evidence of a dose-to-AAD rate response remains to be demonstrated. Further work is required to assess the role of dose and duration on AAD rates and to include diarrhoea using a standardised definition as an outcome measure in randomised controlled trials.

### Changing epidemiology

#### Issues of surveillance

Globally, there are difficulties in surveillance of antimicrobial resistance (AMR) owing to limited laboratory capacity, harmonised diagnostic procedures and a lack of surveillance networks. Mapping AMR in under-resourced countries requires focus on specimen shipping conditions, data standardisation, absence of contamination and adequate diagnostics [34].

When considering available data on *S. pneumoniae* in resource-limited settings, the extent of outpatient penicillin use correlates with the degree of resistance of invasive isolates. In a surveillance study of hospitalised patients in 11 Asian countries, high-level penicillin resistance was much lower than levels of resistance to erythromycin (72.7%) and multi-drug resistance (59.3%). In South Africa, 18% of 20,100 isolates of *S. pneumoniae* identified were resistant to three typical antibiotics. However, it is important to note that AMR data often come from hospitals attended by wealthy patients, introducing bias. Furthermore, it is important that surveillance of pneumococcal pathogens integrates the effects of other public health measures such as conjugate vaccinations [34].

#### Effect of PCV13 vaccine on serotype selection

The widespread use of pneumococcal vaccines is altering the landscape of resistance. Infections and paediatric carriage has been reduced in classically resistant serotypes (14, 6B, 19F, 23F) which are covered by currently available multivalent PCV vaccines. Serogroups 1, 3, 7 and 19 were the most common of the pneumococcal isolates reported to the European Antimicrobial Resistance Surveillance Network (EARS-Net). A large majority of isolates from serogroups 1, 3 and 7 were susceptible to penicillin and macrolides. For serogroup 19, 52% of the isolates had decreased susceptibility to penicillin and/or macrolides [35].

For countries reporting to EARSnet for 2014, serogroups 1 and 19 were the most prevalent (accounting for 13.2 and 12.8% of isolates, respectively), followed by serogroup 7 (11.9%) and serogroup 3 (8.6%). Among the most commonly reported serogroups, dual non-susceptibility to penicillin and macrolides was mainly observed in serogroups 19, 14, and 6 (by order of decreasing percentage). Single non-susceptibility to penicillins was most common in serogroups 19, 14 and 9, and single non-susceptibility to macrolides was most common in serogroups 19, 1, 14 and 6 [35].

The efficacy of pneumococcal conjugate vaccine (PCV13) was assessed by comparing rates of invasive pneumococcal disease in children before and after the introduction of PCV13 in the United States [34]. A time-series model was used to compare the reported incidence of invasive pneumococcal disease (IPD) to that which would have been expected if PCV13 had not replaced PCV7. The authors determined that the overall incidence of IPD declined by 64% (95% CI 59–68), and that IPD caused by PCV13 minus PCV7 serotypes declined by 93% (95% CI 91–94). It was estimated that over 30,000 cases of IPD and 3000 deaths were averted in the first 3 years after introduction of PCV13 [36].

The effectiveness of the PCV13 vaccine was further assessed in a matched case-control study [37]. A total of 722 cases of invasive pneumococcal disease in children aged 2–59 months were identified through active surveillance in 13 sites; 2991 controls were identified in birth registries and matched to cases by age and post code. PCV13 serotype cases (30%) included most commonly identified serotypes 19A (18%), 7F (4%) and 3 (6%). Vaccine effectiveness against all PCV13 serotypes was 86% (95% CI 75.5–92.3), 85.6% (95% CI 70.6–93.5) for serotypes 19A and 96.5% (95% CI 82.7–100.0) for serotype 7F. The effectiveness against serotype 3 (79.5%, 95% CI 30.3–94.8) and antibiotic non-susceptible invasive pneumococcal disease (65.6%, 95% CI 44.9–78.7) was statistically significant. Vaccine effectiveness against all-cause invasive pneumococcal disease was 60.2% (95% CI 46.8–70.3) and was similar in children with (81.4%, 95% CI 45.4–93.6) and without (85.8%, 95% CI 74.9–91.9) underlying conditions.

Invasive pneumococcal isolates recovered from children aged < 5 years through Active Bacterial Core surveillance were analysed before (2008–2009, *n* = 828) and after (2011–2013, *n* = 600) the implementation of the 13-valent pneumococcal conjugate vaccine (PCV13) [36]. PCR/electrospray ionisation mass spectrometry and whole genome sequence (WGS) analysis was used to identify serotypes, resistance features, genotypes, and pilus types. PCV13 targeted all major 19A and 7F genotypes, and decreased antimicrobial resistance, primarily owing to removal of the 19A/ST320 complex. The strain complex contributing most to the remaining β-lactam resistance during 2011–2013 was 35B/ST558. Significant emergence of non-vaccine clonal complexes was not evident. Because of the removal of vaccine serotype strains, positivity for one or both pilus types (PI-1 and PI-2) decreased in the post-PCV13 years 2011–2013 relative to 2008–2009 (decreases of 32–55% for PI-1, and > 95% for PI-2 and combined PI-1 + PI-2). Beta-lactam susceptibility phenotypes correlated consistently with transpeptidase region sequence combinations of the three major penicillin-binding proteins (PBPs) determined through WGS analysis. Other major resistance features were predictable by DNA signatures from WGS analysis. Multilocus sequence data combined with PBP combinations identified progeny, serotype donors and recipient strains in serotype switch events. PCV13 decreased the frequency of all PCV13 serotype clones and concurrently decreased the frequency of strain subsets with resistance and/or adherence features conducive to successful carriage [38].

#### Effect of PCV13 vaccine on disease severity

The link between disease severity and serotype in adults was evaluated [39]. Serotypes covered by the conjugate pneumococcal vaccine (Serotypes 9V, 14, 6B, 18C, 23F, 19F, and 4) were compared to non-vaccine serotypes. No differences were seen in disease severity or associated mortality among patients infected with PCV serotypes, compared with patients infected with non-vaccine serotypes. Invasive pneumococcal disease, older age, underlying chronic disease, immunosuppression and severity of disease were significantly associated with mortality. No association was found between nosocomial infection with invasive serotypes 1, 5, and 7 and mortality. The risk factors meningitis, suppurative lung complications and pre-existing lung disease were significantly associated with disease severity, independent of infecting serotype. Overall, host factors were more important than isolate serotype in determining the severity and outcome of invasive pneumococcal disease in adults [39].

### Duration of treatment: intravenous to oral switch

Few studies are available to inform duration of intravenous antibiotics for children and when it is safe and appropriate to switch to oral antibiotics. Shorter antibiotic courses can potentially affect antimicrobial resistances. The duration and timing of switching antibiotic administration from intravenous to oral in 36 paediatric infectious diseases has been systematically reviewed and recommendations developed [40]. The minimum intravenous and total antibiotic duration required to achieve outcomes similar to or better than those with traditional longer durations were identified. The minimum intravenous antibiotic duration was less than one day, and, for severe or complicated CAP, initial intravenous treatment was recommended based on expert opinion. The criterion for switch to oral antibiotic was clinical improvement. The minimum total antibiotic duration was 3 days for mild CAP and fewer than or equal to 7 days for moderate or severe uncomplicated CAP. Oral antibiotics were deemed acceptable for most children requiring hospital admission.

A Cochrane review examined randomised controlled trials which evaluated the efficacy of a short-course (2–3 days) *vs* a long-course (5 days) of intravenous antibiotic therapy for severe pneumonia in children aged 2–59 months. Children with debilitating disease, HIV infection, very severe pneumonia and nosocomial pneumonia were excluded. A total of 2352 studies were identified, but none fulfilled the inclusion criteria.

In conclusion, the data presented above demonstrates that there is no evidence to recommend amending the current 2014 ‘Revised WHO Classification and Treatment of Childhood Pneumonia at Health Facilities’, either in terms of drug choice, dosing or duration. Recent systematic reviews support the 2014 recommendations and no new trial evidence counters this view. This review has not focussed on recent aetiology studies, or other data on advances in molecular diagnostics (e.g. pneumococcal DNA load) predicting viral or bacterial respiratory infection. A number of CAP amoxicillin trials are underway comparing varying dose and duration regimens, although there has been limited harmonisation as yet of study design. Not all of these trials may be relevant to the LMIC setting. Few trials as yet are including AMR outcomes, making assessment of optimal treatment recommendations at a population level from an AMR perspective currently not possible. There is a major need for further trials to determine the overall optimal duration of treatment of hospitalised children with severe and very severe pneumonia. The optimal choice of antibiotic for oral step-down regimens is also unclear, specifically amoxicillin compared to agents that treat staphylococcal infection (such as amoxicillin clavulanate).

## Funding

This work was supported by Department of Maternal, Newborn, Child and Adolescent Health, World Health Organization.

## Notes on contributors

***Shrey Mathur*** is a researcher and clinician at St George’s, University of London. His research interests include implementation of evidence-based guidance, antibiotic prescribing, health service delivery and global child health.

***Aline Fuchs*** is a researcher, University Children’s Hospital Basel, Switzerland. Her research interests include modelling and simulation in infectious disease and use of antibiotics in neonates.

***Julia Bielicki*** is a researcher in infection and pharmacology at the University Children’s Hospital Basel and at St George’s, University of London and a paediatric infectious disease specialist. Her research interests are making antimicrobial resistance surveillance data accessible for clinical decision-making, clinical trials addressing optimal management of bacterial infections in childhood and antibiotic stewardship.

***Johannes van den Anker*** is the Eckenstein-Geigy distinguished Professor of Paediatric Pharmacology, University Children’s Hospital Basel, Switzerland. His research interests include developmental, neonatal and paediatric pharmacology.

***Mike Sharland*** is Professor of Paediatric Infectious Diseases and Lead Consultant Paediatrician at St George’s Hospital in London. His research interests include optimising the best use of antimicrobials in children, developing the evidence base for paediatric antimicrobials and antimicrobial stewardship.

**Table:** 

A number of emerging themes have been identified:
(1) The optimal dosing recommendation for amoxicillin remains unclear. There are concerns from recent adult pharmacokinetic data about twice-daily dosing in settings of high pneumococcal resistance. Do 250 mg amoxicillin dispersible tablets cover all the paediatric dosing requirements? (2) It is unclear whether amoxicillin or broader-spectrum antibiotics are most commonly being used to treat CAP in different WHO regions. It is difficult to assess the uptake and implementation of the 2014 WHO CAP guidance (3) There remains no globally relevant head-to-head pragmatic trial directly comparing the effectiveness of amoxicillin with an oral cephalosporin and a macrolide in the ambulatory setting (4) The optimal antibiotic management of hospitalised children with severe and very severe pneumonia as well as severe pneumonia in older hospitalised children remains unclear

## Disclosure statement

No potential conflict of interest was reported by the authors.
